# SARS-CoV-2 antibody seroprevalence in Lebanon: findings from the first nationwide serosurvey

**DOI:** 10.1186/s12879-022-07031-z

**Published:** 2022-01-10

**Authors:** Abbas Hoballah, Rana El Haidari, Ghina Siblany, Fadi Abdel Sater, Samir Mansour, Hamad Hassan, Linda Abou-Abbas

**Affiliations:** 1 General Director of Islamic Health Society, Baabda, Lebanon; 2 Department of Research, Islamic Health Society, Baabda, Lebanon; 3 Faculty of Public Health, Lebanese University, Fanar, Lebanon; 4 Laboratory of Molecular Biology and Cancer Immunology (COVID 19 Unit), Faculty of Science, Lebanese University, Hadath, Lebanon; 5 Department of Informatics, Islamic Health Society, Baabda, Lebanon; 6 Ministry of Public Health, Beirut, Lebanon; 7 Medical Care Laboratory Medicine, Faculty of Public Health, Lebanese University, Zahle, Lebanon; 8 Neuroscience Research Center, Faculty of Medical Sciences, Lebanese University, Beirut, Lebanon; 9 Epidemiological Surveillance Program, Ministry of Public Health, Beirut, Lebanon

**Keywords:** SARS-CoV-2 antibody, Seroprevalence, Lebanon, Serosurvey

## Abstract

**Background:**

Lebanon, a small country in the Middle East, remains severely affected by the COVID-19 pandemic. Seroprevalence surveys of anti-SARS-CoV-2 antibodies provide accurate estimates of SARS-CoV-2 infection and hence evaluate the extent of the pandemic. The present study aimed to evaluate the prevalence of SARS-CoV-2 antibodies in Lebanon and to compare the estimated cumulative number of COVID-19 cases with the officially registered number of laboratory-confirmed cases up to January 15, 2021.

**Methods:**

A nationwide population-based serosurvey study was conducted in Lebanon between December 7, 2020, and January 15, 2021, before the initiation of the national vaccination program. The nCOVID-19 IgG & IgM point-of-care (POCT) rapid test was used to detect the presence of anti-SARS-COV-2 immunoglobulin G (IgG) in the blood. Seroprevalence was estimated after weighting for sex, age, and area of residence and adjusting for the test performance.

**Results:**

Of the 2058 participants, 329 were positive for IgG SARS-COV-2, resulting in a crude seroprevalence of 16.0% (95% CI 14.4–17.6). The weighed seroprevalence was 15.9% (95% CI of 14.4 and 17.4). After adjusting for test performance, the population weight-adjusted seroprevalence was 18.5% (95% CI 16.8–20.2). This estimate implies that 895,770 individuals of the general population were previously infected by COVID-19 up to January 15, 2021 in Lebanon. The overall estimated number of subjects with previous SARS-CoV-2 infection was three times higher than the officially reported cumulative number of confirmed cases. Seroprevalence was similar across age groups and sexes (p-value > 0.05). However, significant differences were revealed across governorates.

**Conclusions:**

Our results suggest that the Lebanese population is still susceptible to SARS-CoV-2 infection and far from achieving herd immunity. These findings represent an important contribution to the surveillance of the COVID-19 pandemic in Lebanon and to the understanding of how this virus spreads. Continued surveillance for COVID-19 cases and maintaining effective preventive measures are recommended to control the epidemic spread in conjunction with a national vaccination campaign to achieve the desired level of herd immunity against COVID-19.

**Supplementary Information:**

The online version contains supplementary material available at 10.1186/s12879-022-07031-z.

## Background

**Fig. 1:**
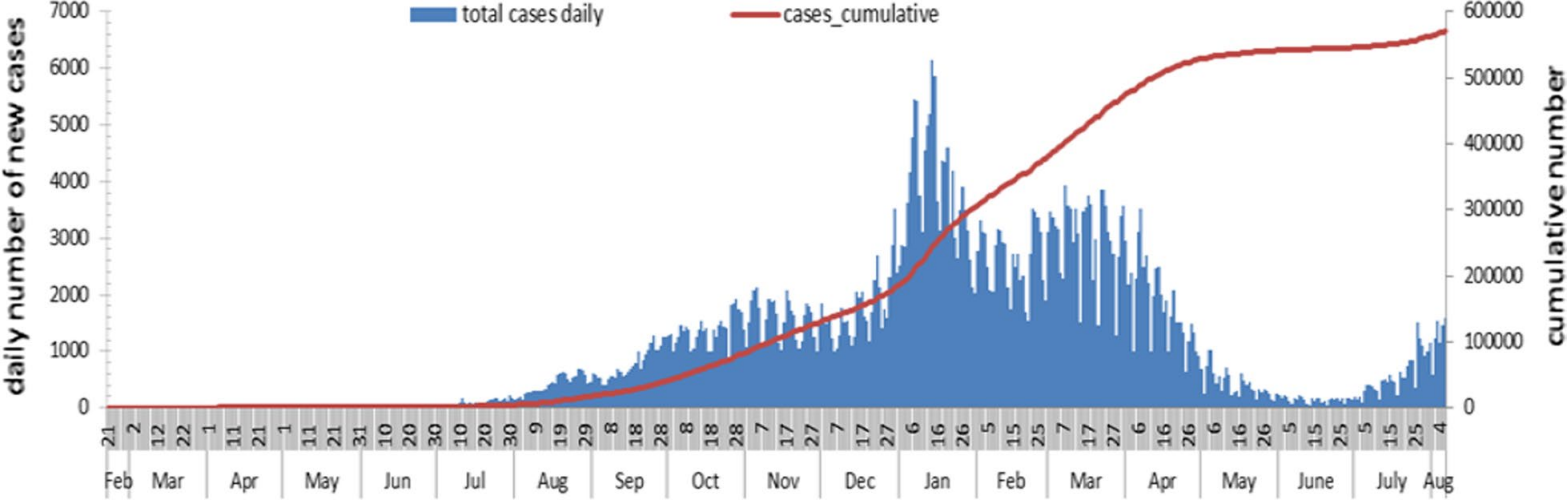
The epidemic curve of laboratory confirmed cases of COVID-19 in Lebanon starting from February 21, 2021 until August 6, 2021 [7]

The global epidemic of coronavirus disease 2019 (COVID-19) has presented a major threat to public health worldwide [1]. The clinical manifestations of infection with the severe acute respiratory syndrome coronavirus 2 (SARS-CoV-2) extend from asymptomatic infections (1.2%), mild cases (80.9%), severe cases (13.8%), to critical illness (4.7%) which can lead to death (2.3%) [2, 3]. Lebanon, a small country in the Middle East, remains severely affected by the COVID-19 pandemic. The first detected COVID-19 case in Lebanon was reported by the Ministry of Public Health (MOPH) on February 21, 2020, after a 45-year-old woman returning from Iran tested positive. Immediately, the patient and her traced contacts were quarantined. On February 22, Lebanon shut down public transport and banned flights to countries that had experienced exponential growth patterns of COVID-19, including Iran, Italy, China, and South Korea. A few days later, several measures were imposed to reduce the spread of the infection, including enforced partial curfew hours, as well as closing daycare centers, schools, universities, malls, restaurants, tourist sites, public gardens, nightclubs, pubs, gyms, and theaters. By mid-March, with the number of confirmed cases climbing to 99, the Lebanese government declared a state of health emergency and imposed a 2-week full lockdown including the closure of the Beirut Rafic Hariri International Airport, as well as sea and land ports except for diplomatic missions and cargo aircrafts. On April 27, a phased reopening was initiated after a decrease in the average growth factor per week from 4.6 in week 2 (February 28–March 5) to less than 1 in week 6 (March 27–April 2). On July 1st, 2021, the Lebanese government decided to reopen Beirut Airport. However, at the end of July, a few weeks after reopening the airport, there was a significant rise in the number of COVID-19 patients in various districts. Thus, community transmission was declared. Exploration of the epidemic spread using data provided by the MOPH until June 30, 2020 revealed that the initial value of the reproductive transmission factor (R0) was remarkably high at R0 = 5.6. However, it significantly decreased to R1 = 0.52 after 32 days, before rising moderately to 1.1 after 90 days following a partial relaxation of social distancing measures [4]. After the devastating explosion that occurred at the Beirut Port on August 4, 2020, the daily number of new cases increased to 500–1000 between August and mid-September, then to 1000–2000 from Mid-September to December. In January 2021, the country experienced its first large wave when the number of daily confirmed cases climbed up reaching 6000 on 14 January 2021 [5]. After this peak, the curve went down following lockdown measures, with a relative increase in March 2021. Between April and June 2021, a continuous decline in the confirmed daily cases occurred. However, in July 2021, the daily number of new cases started a new increase, approaching 1500 per day. As of 6 August 2021, the time-varying reproduction number, calculated as the average number of secondary infections caused by a single infected person at a given time, was estimated as 1.25 [confidence interval (CI) of 1.17–1.34] suggesting that the number of new infections and the rate of transmission were increasing [6]. Figure 1 illustrates the epidemic curve of laboratory confirmed cases in Lebanon starting February 21 and up to August 6, 2021 [7].

Diagnostic testing for SARS-CoV-2 infections, including rapid antigen testing or polymerase chain reaction (PCR)-based viral RNA testing, is a critical component to the overall prevention and control strategy for COVID-19 [8]. Mass screening were undertaken by the Lebanese government to contain the spread of the epidemic, including testing the contacts of positive cases, people possibly exposed by travel-related affairs, or individuals showing symptoms [9]. Nevertheless, the majority of the population remained untested due to several factors. To name a few, we had limited availability and accessibility of testing, high cost of the reverse transcription-polymerase chain reaction (RT-PCR) test, stigma or simply the lack of perceived need especially among mild and asymptomatic cases [9]. Thus, the number of confirmed SARS-CoV-2 infections is likely to be significantly underestimated. Consequently, epidemiological surveillance of confirmed COVID-19 cases might catch only a proportion of all SARS-COV-2 infections, and the epidemic curves might not be representative of the disease true burden in the community.

Recently, the World Health Organization (WHO) has recommended conducting seroprevalence surveys to determine the presence of anti-SARS-CoV-2 antibodies in blood samples and evaluate the extent of the COVID-19 pandemic [10]. Thus, several nationwide studies have been conducted to estimate the seroprevalence of SARS-CoV-2 antibodies (IgG and/or IgM) among the general population [11–18]. Data from these studies can be used as a reference in the evaluation of the implemented preventive measures and in optimizing COVID-19 vaccine distribution.

At the time of writing, no seroprevalence studies were conducted for refining estimates of COVID-19 cases and transmission and hence, evaluating the pandemic impact on the Lebanese society. Thus, the present study objectives were to estimate the prevalence of SARS-CoV-2 antibodies in a representative large sample of citizens and to compare the estimated number of cases with the officially registered number of laboratory confirmed cases reported by the MOPH up to until January 15, 2021.

## Methods

### Study design and population

A nationwide population-based serosurveillance study was conducted in Lebanon between December 7, 2020 and January 15, 2021, before the initiation of the national vaccination program. The Lebanese Republic, a country in the Levant region of western Asia and the transcontinental region of the Middle East, is composed of eight governorates (Beirut, Mount Lebanon, North Lebanon, Akkar, Baalbek El-Hermel, Bekaa, Nabatieh, and South Lebanon) including 24 districts.

All residents who were deemed to be asymptomatic for SARS-CoV-2 infection at the time of data collection were eligible to participate. The exclusion criteria included patients confirmed with SARS-CoV-2 infection, individuals under quarantine, and patients presenting symptoms suggestive of SARS-CoV-2 infection including cough, fever, body aches, and shortness of breath [19], as well as those who refused to give informed consent.

### Sample size

The sample size was calculated using the Epi Info™ tool (Center for Disease Control, Atlanta, GA, USA available from http://wwwn.cdc.gov/epiinfo/) [20]. The sample size was calculated based on an estimated COVID-19 prevalence of 16% based on a previous study result [21], a confidence interval of 95%, a maximum allowable error in the prevalence of 1%, a non-response rate of 10%, and a Lebanese population size of 4,842,000 inhabitants based on the latest Lebanese census data [22]. The minimum required sample size was 2170. The total number of participants who accepted to participate was 2058, making the response rate 95%.

### Sampling procedure

A stratified random sampling method was used in this survey to select a representative sample from across Lebanon’s eight governorates. The number of participants in each governorate was calculated using a probability-proportion-to-size sampling method based on the data provided by the Central Administration of Statistics (CAS) in 2019 (Table 1) [22]. In each governorate, six villages were randomly selected from a list provided by the CAS except for North Lebanon and Akkar governorates where only two and three villages had been selected respectively, giving a total of 41 villages. Within each village, a random starting point was selected by the research team who visited a fixed number of households to enroll one person from each. In case of participation refusal or no one at home, the team proceeded to the next house and completed the survey until the required number of households was enrolled from each village. A table of the number of participants according to Lebanese governates and villages has been provided in the Additional file 1.

**Table 1:** Regional distribution of the study population

Governorates	Population size	Percentages of population	Estimated sample size	Collected sample
Beirut	342,000	7	153	162
Mount Lebanon	203,3000	42	911	925
North Lebanon	638,000	13	286	163
Akkar	324,000	7	145	119
Bekaa	298,000	6	134	143
Baalbek El-Hermel	245,000	5	110	112
South Lebanon	584,000	12	262	269
Nabatieh	379,000	8	170	165
Lebanon	4,842,000	100	2170	2058

### Data collection

Fieldwork was conducted by professional teams from the Islamic Health Society who were responsible for data collection and blood sampling. All fieldwork members underwent infection-control training and were provided with personal protective equipment. During the visit, the interviewer explained the purpose of the study to potential participants. After obtaining informed consent, the interviewers used a paper-based survey to collect data on sociodemographic characteristics (age, sex, and residency), clinical information about comorbidities, history of positive PCR testing for SARS-CoV-2 and previous symptoms suggestive of COVID-19. Upon sample collection, trained laboratory personnel analyzed the serological test, and the participants were promptly informed about the test results thereafter. Positive cases, as well as cases with suggestive symptoms of COVID-19, were reported to the MOPH surveillance system. Data entry was performed and double-checked by researchers not involved in the data collection process. Then, anonymized data were saved on a secure server.

### Serological analyses

Serological testing was conducted by the point-of-care test (nCOVID-19 IgG & IgM POCT; Corso Vittorio Emanuele II,15,20122, Milano, ITALIA) applied as per the manufacturer’s instructions by pricking finger and applying a drop of blood to a test strip. This test is a lateral-flow immunochromatographic assay for qualitative differentiation between IgG and IgM against the receptor binding domain of SARS-CoV-2 spike (S) protein. This is a simple cassette-based test that works with just 10–20 µl of serum, plasma or whole blood. This serum test can be used by a health care professional outside the laboratory settings and yields results in ten minutes. The manufacturer reported clinical sensitivity of 90·0% and a specificity of 96.3%. An in-house validation study, using a set of sera from 25 negative controls and 25 RT-PCR-confirmed SARS-CoV-2 infection cases was conducted to estimate test performance. This validation pilot study revealed sensitivity of 92.3% and a specificity of 100%.

### Ethical statement

The protocol of the study was approved by the research ethics committee of the Islamic Health Society (reference number: 1687.24251). Researchers and field workers conducted the study according to the research ethics guidelines laid down in the Declaration of Helsinki of the World Medical Association Assembly [23]. Participation in the study was voluntary and an informed consent was obtained from each participant. All the necessary measures to safeguard the participants’ anonymity and the confidentiality of information were respected.

### Statistical analysis

Data analyses were performed using the statistical software SPSS version 26.0 (IBM Corp. Released 2019.IBM SPSS Statistics for windows, Version 26.0. Armonk, N.Y: IBM Corp). Descriptive statistics were reported using means along with standard deviations (SD) for continuous variables and frequency with percentages for categorical variables. The seroprevalence of IgG antibodies against SARS-COV-2 with 95% CI was estimated as the proportion of individuals who had a positive result in the IgG band of the point-of-care test. We also calculated seroprevalences by age group, sex, area of residence and SARS-CoV-2 infection-related characteristics of study participants. In addition, seroprevalences were weighted by sex, age and area of residence to compensate for differences between the sample and the target profile by using the data provided by the Central Administration of Statistics (CAS) [13].

The weighted seroprevalences were further adjusted for the sensitivity and specificity of the assay using the formula [24]:

$${\text{P corrected}} = {{\left( {{\text{P}} + {\text{Sp}} - {1}} \right)} \mathord{\left/ {\vphantom {{\left( {{\text{P}} + {\text{Sp}} - {1}} \right)} {\left( {{\text{Se}} + {\text{Sp}} - {1}} \right)}}} \right. \kern-\nulldelimiterspace} {\left( {{\text{Se}} + {\text{Sp}} - {1}} \right)}},$$

where P is the weighted prevalence, Se is the sensitivity of the assay, and Sp is the specificity of the assay. The stratum seroprevalence and 95% confidence intervals (CIs) of SARS-CoV-2 seropositivity were calculated.

The estimated number of subjects with previous COVID-19 was calculated by multiplying the seropositivity rates for each area of residence by the estimated population size. Results were compared with official numbers and rates of reported cases issued by the Lebanese Ministry of Public Health as of January 15, 2021 [5]. Ratios between population-wide seroprevalence estimates and the cumulative incidence of confirmed SARS-CoV-2 infections were calculated in the overall population and across governorates. The infection fatality ratio (IFR) was also calculated on the basis of the cumulative number of confirmed SARS-CoV-2 infection deaths as of January 15, 2021 divided by the number of infections inferred from seroprevalence data. Statistical significance was set at p-value < 0.05.

## Results

### Characteristics of the study population

Table 2 shows the demographic characteristics of participants. Between December 7, 2020 and January 15, 2021, a total of 2058 participants were included in the study. The mean participants age was 37.8 ± 16.7 years. Of the total, 1207 (58·6%) of participants were women; 270 (13.1%) had at least one comorbidity condition, 60 (2.9%) reported a history of positive PCR testing for SARS-CoV-2 with 71.6% of them have had COVID-19 symptoms.

**Table 2:** Characteristics of the study participants

Characteristics	N	%
Overall	2058	
Age mean (SD)	37.8 ± 16.7	
Age groups, years		
≤ 19	293	14.2
20–29	403	19.6
30–39	366	17.8
40–49	360	17.5
50–59	320	15.5
≥ 60	200	9.7
Missing data	116	5.6
Sex		
Male	851	41.4
Female	1207	58.6
Comorbidities		
Yes	270	13.1
No	1432	69.6
Don’t know	356	17.3
History of positive PCR testing for SARS-CoV-2		
Yes	60	2.9
No	1642	79.8
Don’t know	356	17.3
Presence of symptoms for who those who had a history of positive PCR^a^		
Yes	43	71.6
No	17	28.4

*N* number; *%* percentage; *SD* standard deviation

^a^Presence of at least symptoms for participants who had a history of positive PCR testing for SARS-CoV-2

### Prevalence of SARS-CoV-2 antibodies

Table 3 presents the seroprevalence of SARS-CoV-2 antibodies in the overall sample and stratified by sociodemographic characteristics. Of the 2058 participants, 329 were positive for IgG SARS-CoV-2 resulting in a crude seroprevalence of 16.0% (95% CI of 14.4–17.6). Crude seroprevalence did not vary by sex (15.6% versus 16.6% for males and females respectively, p-value = 29.0) but did vary by age group from 11.7% (95% CI of 8.6–14.8) among adults aged 20–29 years old to 19.2% (95% CI of 15.1–23.3) among their counterparts aged 40–49 years (p-value = 08.0). Crude seroprevalence varied geographically from 29.5 (95% CI of 21.1–37.9) in Baalbek El-Hermel to 6.1% (95% CI of 2.4–9.8) in Nabatieh (p-value < 0.0001).

**Table 3:** Proportion positive with SARS-CoV-2 IgG

Characteristics	N	N Positive	Crude	p-value	Weighted*	Adjusted for test performance	p-value
Overall	2058	329	16.0(14.4;17.6)		15.9(14.4;17.4)	18.5(16.8;20.2)	
Sex				0.29			0.60
Male	1207	188	15.6(13.6;17.6)		15.6(13.6;17.5)	18.0(15.8;20.2)	
Female	851	141	16.6(14.1;19.1)		16.4(14.0;18.7)	19.2(16.6;21.8)	
Age groups (years)				0.08			0.08
≤ 19	293	45	15.4(11.3;19.5)		15.0(11.2;18.8)	17.8(13.2;22.2)	
20–29	403	47	11.7(8.6;14.8)		12.2(9.2;15.2)	13.5(10.2;16.8)	
30–39	366	63	17.2(13.3;21.1)		17.1(13.5;20.7)	19.9(15.8;24.0)	
40–49	360	69	19.2(15.1;23.3)		19.0(15.2;22.8)	22.2(17.9;26.5)	
50–59	320	52	16.3(12.3;20.3)		15.7(11.9;19.5)	18.8(14.5;23.1)	
≥ 60	200	37	18.5(13.1;23.9)		18.6(13.5;23.7)	21.4(15.7;27.1)	
Governorates				< 0.0001			< 0.0001
Baalbek El-Hermel	112	33	29.5(21.1;37.9)		29.8(21.0;38.6)	34.1(25.3;42.9)	
North Lebanon	163	30	18.4(12.5;24.3)		18.2(12.2;24.2)	21.3(15.0;27.6)	
Bekaa	143	26	18.2(11.9;24.5)		18.0(11.5;24.5)	21.0(14.3;27.7)	
Mount Lebanon	925	167	18.1(15.6;20.6)		18.1(15.8;20.4)	20.9(18.3;23.5)	
South Lebanon	269	34	12.6(8.6;16.6)		12.6(9.5;15.7)	14.6(10.4;18.8)	
Akkar	119	13	10.9(5.3;16.5)		11.2(5.2;17.2)	12.6(6.6;18.6)	
Beirut	162	16	9.9(5.3;14.5)		10.1(5.3;14.9)	11.4(6.5;16.3)	
Nabatieh	165	10	6.1(2.4;9.8)		6.1(2.2;10.0)	7.0(3.1;10.9)	
Comorbidities				0.25			0.39
Yes	270	39	14.4(10.2;18.6)		16.2(12.1;20.3)	16.6(12.2;21.0)	
No	1432	233	16.3(14.4;18.2)		14.1(12.4;15.8)	18.8(16.8;20.8)	
History of positive PCR testing for SARS-CoV-2				< 0.0001			< 0.0001
Yes	60	38	63.3(51.1;75.5)		63.2(51.7;74.7)	73.3(62.1;84.5)	
No	1642	234	14.3(12.6;16.0)		14.1(12.5;15.7)	16.5(14.7;18.3)	

*N* number; *%*: percentage; *IgG* immunoglobulin G; *CI* confidence interval

*Weighted by age group, sex, and governorates

**Fig. 2:**
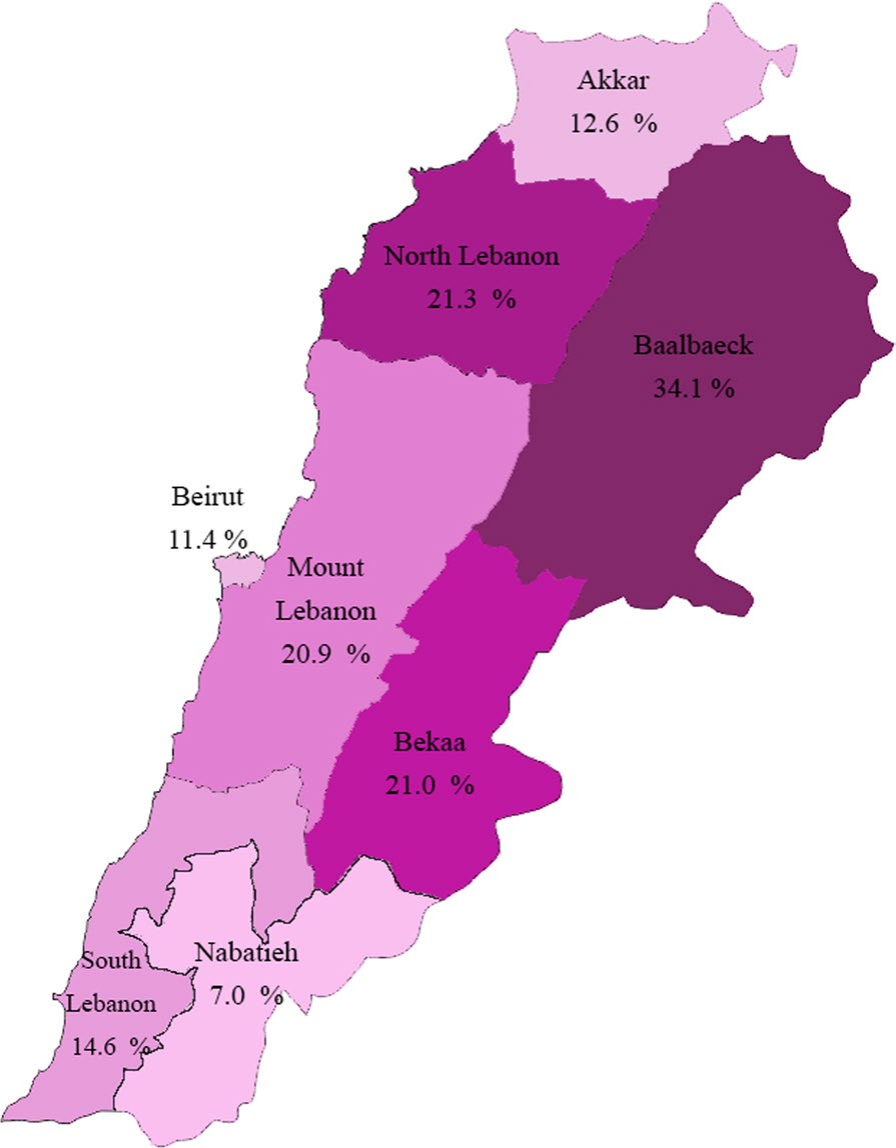
Nationwide seroprevalence of SARS-CoV-2 in Lebanon

Weighted seroprevalences by demographic characteristics were calculated to match the 2019 Lebanese population census data. Overall, the weighed seroprevalence was 15.9% (95% CI of 14.4 and 17.4) with 16.4% (95% CI of 14.0 and 18.7) among females and 15.6% (95% CI of 13.6 and 17.5) among males. The highest seroprevalence was observed in individuals aged between 40–49 years (19.2%, 95% CI of 15.1 and 23.3) and the lowest among those aged 20–29 (12.2%, 95% CI of 9.2 and 15.2). Estimates varied markedly across provinces with the highest positive seroprevalence in Baalbek El-Hermel (29.8%, 95% CI of 21.0–38.6), followed by North Lebanon (18.2%, 95% CI of 12.2 and 24.2), Bekaa (18.0%, 95% CI of 11.5 and 24.5), Mount Lebanon (18.1%, 95% CI of 15.8 and 20.4), South Lebanon (12.6, 95% CI of 9.5–15.7), Akkar (11.2%, 95% CI of 5.2 and 17.2), Beirut (10.1%, 95%CI of 5.3–14.9) and Nabatieh (6.1%, 95% CI of 2.2–10). The prevalence of IgG antibody among individuals who had at least one comorbidity (14.4%, 95% CI of 10.2 and 18.6) was lower compared to individuals with no comorbidities (16.3%, 95% CI of 14.4–18.2). Finally, the seroprevalence estimate in individuals who reported a history of positive PCR testing for SARS-CoV-2 was considerably higher (63.3%, 95% CI of 51.1–75.5) compared to individuals without history of positive PCR 14·3% (95% CI of 12.6–16.0). After adjusting for test performance, the population weight-adjusted seroprevalence was 18.5% (95% CI of 16.8–20.2). Across provinces, the highest positive seroprevalence was in Baalbek El-Hermel (34.1%, 95% CI of 25.3–42.9), followed by North Lebanon (21.3%, 95% CI of 15.0 and 27.6), Bekaa (21.0%, 95% CI of 14.3 and 27.7), Mount Lebanon (20.9%, 95% CI of 18.3 and 23.5), South Lebanon (14.6, 95% CI of 10.4–18.8), Akkar (12.6%, 95% CI of 6.6 and 18.6), Beirut (11.4%, 95%CI of 6.5–16.3) and Nabatieh (7.0%, 95% CI of 3.3–10.9) (Fig. 2). IFR inferred from our seroprevalence data was estimated at 0.21%.

### Comparison of seroprevalence results with reported cases by the Lebanese Ministry of Public Health

As of January 15, 2021, the officially registered cumulative confirmed COVID-19 disease cases were 249,158 [5]. Our estimated overall seroprevalence of 18.5% implies that 895,770 were previously infected by COVID-19. As a result, the overall estimated number of subjects with previous COVID-19 was three times higher than the cumulative number of confirmed cases. In the governorates, the seroprevalence estimate was higher than the cumulative reported case by a factor ranging between 1.6 to 13.9 times with the highest one reported in Akkar governorate. Regarding the percentage of underestimated cases, overall 13.4% of cases in the general population were underestimated. The highest percentages of underestimated cases were found in Baalbek El-Hermel (28.2%) followed by North Lebanon (16.5%), Bekaa (15.8%), Mount Lebanon (15.7%), Akkar (11.7%), South Lebanon (11.5%), and the lowest percentages were reported in Nabatieh (4.6%) and Beirut (4.5%) (Table 4).

**Table 4:** Comparison of SARS-CoV-2 IgG results with reported cases by the Lebanese Ministry of Public Health

Governorates	Total Population	Weight-adjusted Seroprevalence %	Cumulative number of COVID-19*	Estimated number of subjects with previous COVID-19** n	Ratio of estimated number to Cumulative number of COVID-19	Percentage of underestimated cases*** % (95%CI)
Beirut	342,000	11.4	23,749	38,988	1.6	4.5(1.3;7.7)
Mount Lebanon	2,032,000	20.9	105,716	424,688	4.0	15.7(13.4;18.0)
Baalbek El-Hermel	245,000	34.1	14,417	83,545	5.8	28.2(19.9;36.5)
Bekaa	298,000	21.0	15,602	62,580	4.0	15.8(9.8;21.8)
North Lebanon	638,000	21.3	30,805	135,894	4.4	16.5(10.8;22.2)
Akkar	324,000	12.6	2,930	40,824	13.9	11.7(5.9;17.5)
South Lebanon	584,000	14.6	18,242	85,264	4.7	11.5(7.7;15.3)
Nabatieh	379,000	7.0	8910	26,530	3.0	4.6(1.4;7.8)
Lebanon	4,842,000	18.5	249,158	895,770	3.6	13.4 (11.9;14.9)

*MOPH* Lebanese Ministry of Public Health; *IC* confidence interval

*Cumulative number reported by MOPH until January 15, 2021

**Estimated number of subjects with previous COVID-19 calculated by multiplying the seropositivity rates for each governorate by the estimated population size

***Percentage of underestimated cases = (Estimated total number of seropositive − registered cases up to 15/1/2021)*100/total population

## Discussion

To the best of our knowledge, this is the first nationwide seroprevalence survey describing the prevalence of SARS-CoV-2 antibodies in the Lebanese population and comparing the estimated number of cases with the officially registered number of laboratory confirmed cases reported by the MOPH until January 15, 2021. Our results showed that the prevalence of SARS-CoV-2 antibodies was around 18% with an estimated cumulative total number of 895,770 COVID-19 cases. The overall estimated number of subjects with previous COVID-19 was three times higher than the officially reported cumulative number of confirmed cases. No significant differences in SARS-CoV-2 seropositivity regarding sex and age subgroups were found. However, significant differences were revealed across governorates.

The results of the current study revealed an overall estimated weighted seroprevalence of 18.5% which implies that the Lebanese general population is still susceptible to SARS-CoV-2 infection and has not reached the desired level of herd immunity (60–70%). This low level could explain the ongoing wave epidemic intensity at the time of the study. A nationwide serosurvey conducted in India during the same period (December 2020 and January 2021) reported a higher seroprevalence estimate of 24% [18]. In addition, a Systematic review, summarizing data from January 1, 2020 to December 31, 2020 and including 9.3 million participants from 74 countries, has also revealed wide discrepancies in SARS-CoV-2 antibodies seroprevalences across geographic regions [25]. These discrepancies could be explained by the variation in the community spread of the virus and the applied preventive measures in each country.

Although earlier studies reported that women were less vulnerable to SARS-CoV-2 infection and less likely to show signs of COVID-19 compared to men [26–30], our findings were consistent with more recent studies that revealed no substantial differences in seroprevalence rates by sex [31, 32]. Regarding age group, our study didn’t reveal any difference in seropositivity. This finding was inconsistent with a previous study which had found that people in the age group of 30–69 years had higher odds of seropositivity as compared to the younger population [32]. However, as expected, seropositivity varied significantly between governorates with the highest level found in Baalbek El-Hermel (weighed seroprevalence of 34.1% with 95% CI of 25.3–42.9) and the lowest one in Nabatieh (weighed seroprevalence of 7.0% with 95% CI of 3.1–10.9). These variations could be attributed to many factors, including variation in the community transmission across governorates, mitigation efforts, cultural practices, and the implementation of preventive measures.

Consistent with previous reports [25, 26], we found a significant difference between the officially registered number of COVID-19 cases and the extrapolated cumulative number of cases based on seroprevalence data. Approximately threefold more infections occurred in seroprevalence data what were ascertained by confirmed case counts, suggesting an underestimation of the epidemic extent in Lebanon. This ratio is relatively low compared to the ones reported in previous studies which ranged from 6.7-fold in South Asia to 602.5-fold in Sub-Saharan Africa [25]. Our findings also revealed that seroprevalence to cumulative case ratios varied substantially between governorates from 1.6-fold in Beirut to 13.9-fold in Akkar governorate. These variations could be explained by the limited availability and accessibility of testing in the socioeconomically deprived governorates such as Akkar and Baalbek-Hermel, as well as the increased number of undetected asymptomatic infections. Region-specific ratios are helpful in identifying areas that may be receiving potentially insufficient levels of testing.

As of January 15, 2021, the IFR calculated from officially registered COVID-19 cases and deaths was 0.75% [5]. However, the inferred IFR from our seroprevalence (0.21%) tended to be much lower. This comes in consistency with the study of Ioannidis [27]. Consequently, ongoing seroprevalence survey might be a better approach to complement surveillance data and to provide a more accurate picture of the pandemic actual burden.

It is worth mentioning that the serological surveys are the best tool to determine the spread of an infectious disease, particularly in the presence of asymptomatic cases or incomplete ascertainment of those with symptoms [32]. Different studies have reported a decrease of SARS-CoV-2 antibodies in the months following infection [15, 33, 34]. Most convalescent people who recover from SARS-CoV-2 do not have high levels of neutralizing activity [35]. This possible loss of immunity over time must be considered when interpreting seroprevalence studies for SARS-CoV-2.

Our study has several strengths: This is the first national study concerning seroprevalence of SARS-CoV-2 in Lebanon. In this respect, our study provides new information about the true number of COVID-19 cases in our country. The sample was designed to be representative in terms of governorates. Additionally, the overall prevalence estimates were adjusted not only for sex and age groups, but also for test performance to make the findings representative of the study population. The non-response rate at the household level was relatively low compared to other population-based survey [15, 17, 30–36]. Finally, Individuals with current SARS-CoV-2 symptoms were excluded to avoid an inflated proportion of individuals with negative tests which could underestimate the seroprevalence. Despite these strengths, our study has some limitations that should be noted. For instance, the collected data was based on self-reported information which makes it prone to the social desirability and recall biases. In addition, some of the participants were unaware or refused to answer some of the survey questions which may result in non-response bias. However, it is expected that the weighting controlled the non-response bias of the present survey. Considering the specificities of used tests and the cross-reactivity with other viruses such as endemic non-SARS-CoV-2, we cannot completely rule out the possibility of false results that might overestimate the seroprevalence among the study population. Finally, the cumulative number of officially laboratory-confirmed COVID-19 cases in Lebanon have increased substantially since the completion of the serosurvey, meaning that, the results reported in this study does not represent the true seroprevalence at the time of writing and publication of this study. Further studies are needed to investigate other sociodemographic characteristics affecting SARS-COV-2 seroprevalence such occupation (Health worker/Frontline workers) and economic classes.

## Conclusions

Our results suggest that at the time of writing the study, the Lebanese population was still susceptible to SARS-CoV-2 infection and far from achieving herd immunity. Infection rates based on surveillance data considerably underestimated the actual burden of the SARS-CoV-2 infection in Lebanon. Our findings represent an important contribution to the surveillance of the SARS-CoV-2 infection in Lebanon and to the understanding of how this virus spreads. As the number of SARS-CoV-2 infection cases is still growing rapidly, seroprevalence surveillance should be sustained and is necessary to gain insight into the transmission dynamics of SARS-CoV-2 infection. Furthermore, continued surveillance for COVID-19 cases and maintaining effective preventive measures are recommended to control the epidemic spread in conjunction with a national vaccination campaign to achieve the desired level of herd immunity against COVID-19. Last but not the least, additional investigations are required, especially longitudinal serological studies using the gold standard test, to monitor the extent of the pandemic and determine the persistence of antibody-mediated immunity.

## Supplementary Information

**Additional file 1: Table SA.** The number of participants according to Lebanese governates and villages.

## Data Availability

The datasets used and/or analyzed during the current study are available from the corresponding author on reasonable request.

## Abbreviations

CAS: Central Administration of Statistics
CI: Confidence interval
COVID-19: Coronavirus Disease 2019
IFR: Infection fatality ratio
IgG: Immunoglobulin G
MOPH: Ministry of Public Health
RT-PCR: Reverse Transcription Polymerase Chain Reaction
SARS-CoV-2: Severe acute respiratory syndrome coronavirus 2
SD: Standard deviation
WHO: Word Health Organization
