# Supplementary material for: SARS-CoV-2 antibody seroprevalence in Lebanon: findings from the first nationwide serosurvey
Source: BMC Infect Dis. 2022 Jan 10;22:42. doi: 10.1186/s12879-022-07031-z (PMC8744021; doi:10.1186/s12879-022-07031-z)
Supplement: Supplementary file 1 — Additional file 1: Table SA. The number of participants according to Lebanese governates and villages. [file 12879_2022_7031_MOESM1_ESM.pdf]

Table A. The number of participants according to Lebanese **governorates and villages**

Governates	Villages	Number of segments	Estimated sample size	Number of participants	Response rate
Beirut	Ras nabea	4	26	26	100
	Nwayreh	3	26	27	104
	Zkak lblat	4	26	26	100
	Khandaa ghamea	1	26	26	100
	tarea jdideh	4	30	30	100
	Mala	3	26	27	104
	Total	19	153	162	106
Mount Lebanon	Haret Hreik	8	151	152	101
	Jounieh	10	151	153	101
	Jiyeh	10	152	155	102
	Amrousiyeh	6	152	155	102
	Chouweifat	6	152	155	102
	Maten Aala	10	152	155	102
	Total	50	911	925	102
Bekaa	Machghara	4	23	25	109
	Libeya	4	23	25	109
	Sohmor	4	23	25	109
	rachya	3	23	25	109
	Hasbya	3	22	21	95
	Karoon	3	20	20	100
	Total	23	134	143	107
Baalbek El-Hermel	Hermel	4	20	20	100
	Britel	4	10	10	100
	Bouday	4	15	15	100
	Zahle	4	20	20	100
	bet shema	4	15	17	113
	Baalbek	4	30	30	100
	Total	24	110	112	102
South Lebanon	Jizin	3	42	42	100
	Hosh	5	43	43	100
	Sour	4	45	50	111
	Ghaziyeh	3	45	44	98
	Sarafand	4	42	45	107
	Borj shmele	3	45	45	100
	Total	22	262	269	103
Nabatieh	Ayneta	4	28	28	100
	Taybeh	4	28	28	100
	Aytaroun	3	28	28	100
	Bent Jbeil	4	30	30	100
	Kfarkila	3	28	28	100
	Idayse	3	28	23	82
	Total	21	170	165	97
North Lebanon	Batroun	4	95	60	63
	Bcharre	4	95	50	53
	Bnahran	3	96	53	55
	Total	11	286	163	57
Akkar	Habchit	3	73	69	95
	Akkar	5	72	50	69
	Total	8	145	119	82
